# Pea proteins oral supplementation promotes muscle thickness gains during resistance training: a double-blind, randomized, Placebo-controlled clinical trial vs. Whey protein

**DOI:** 10.1186/s12970-014-0064-5

**Published:** 2015-01-21

**Authors:** Nicolas Babault, Christos Païzis, Gaëlle Deley, Laetitia Guérin-Deremaux, Marie-Hélène Saniez, Catherine Lefranc-Millot, François A Allaert

**Affiliations:** National Institute for Health and Medical Research, (INSERM), unit 1093, Cognition, Action and Sensorimotor Plasticity, Dijon, France; Centre for Performance Expertise, UFR STAPS, Dijon, France; Roquette, Lestrem, France; Chair of Medical Evaluation ESC, Dijon, France; CEN Nutriment, Dijon, France; Faculté des Sciences du Sport, Université de Bourgogne, BP 27877, 21078 Dijon Cedex, France

**Keywords:** Muscle strength, Biceps brachii, Muscle thickness, Feeding, Hypertrophy, Nutralys

## Abstract

**Background:**

The effects of protein supplementation on muscle thickness and strength seem largely dependent on its composition. The current study aimed at comparing the impact of an oral supplementation with vegetable Pea protein (NUTRALYS®) vs. Whey protein and Placebo on biceps brachii muscle thickness and strength after a 12-week resistance training program.

**Methods:**

One hundred and sixty one males, aged 18 to 35 years were enrolled in the study and underwent 12 weeks of resistance training on upper limb muscles. According to randomization, they were included in the Pea protein (n = 53), Whey protein (n = 54) or Placebo (n = 54) group. All had to take 25 g of the proteins or placebo twice a day during the 12-week training period. Tests were performed on biceps muscles at inclusion (D0), mid (D42) and post training (D84). Muscle thickness was evaluated using ultrasonography, and strength was measured on an isokinetic dynamometer.

**Results:**

Results showed a significant time effect for biceps brachii muscle thickness (*P* < 0.0001). Thickness increased from 24.9 ± 3.8 mm to 26.9 ± 4.1 mm and 27.3 ± 4.4 mm at D0, D42 and D84, respectively, with only a trend toward significant differences between groups (*P* = 0.09). Performing a sensitivity study on the weakest participants (with regards to strength at inclusion), thickness increases were significantly different between groups (+20.2 ± 12.3%, +15.6 ± 13.5% and +8.6 ± 7.3% for Pea, Whey and Placebo, respectively; *P* < 0.05). Increases in thickness were significantly greater in the Pea group as compared to Placebo whereas there was no difference between Whey and the two other conditions. Muscle strength also increased with time with no statistical difference between groups.

**Conclusions:**

In addition to an appropriate training, the supplementation with pea protein promoted a greater increase of muscle thickness as compared to Placebo and especially for people starting or returning to a muscular strengthening. Since no difference was obtained between the two protein groups, vegetable pea proteins could be used as an alternative to Whey-based dietary products.

**Trial registration:**

The present trial has been registered at ClinicalTrials.gov (NCT02128516).

## Background

Amino acids accumulate in the sarcoplasm in the hours following exercise, especially of weight-training type [1]. Such accumulation undoubtedly creates favorable conditions to protein synthesis. Indeed, increased protein synthesis (i.e., muscle hypertrophy) is observed immediately after resistance exercises [2-4] as a result of a positive protein net balance; the difference between muscle fiber catabolism and anabolism. Under these conditions, any nutritional modification that could increase protein accretion in the muscle would maximize resistance training effects by enhancing muscle anabolism. In particular, it has now been well demonstrated that protein consumption after exercise shifts the balance in favor of muscle protein synthesis [5]. Taken altogether, these data clearly demonstrate the great interest of an association between amino acids supplementation and resistance training.

Composition of supplements may play a key role in influencing net protein balance since previous studies have revealed that only essential amino acids (EAA) could stimulate muscle protein synthesis [6]. Furthermore, protein type, and not simply its amino acid composition, can differentially modulate protein synthesis depending on digestion kinetics. For example, in a recent review [1], it was speculated that whey, with high leucine content and rapid digestion kinetic [7], may favor muscle protein synthesis while casein, with a slower digestion kinetic [7], may improve muscle net balance by inhibiting muscle protein breakdown. In addition to increases in muscle mass, functional adaptations, such as strength or fatigue, are also obtained after EAA supplementation [8,9]. For example, Vieillevoye et al. [10] found increases in lower body strength with EAA supplementation while no modification was obtained with placebo.

Different supplements’ compositions could easily be obtained with different nutrients. For example, NUTRALYS® is a protein isolate obtained from pea (*Pisum sativum*) containing 85% of proteins and particularly rich in essential branched-chain amino acids (BCAA; leucine, isoleucine and valine) known to play an important role in muscle protein synthesis [11]. Studies have shown that an increased plasma concentration in leucine favors muscle protein synthesis and that its action on muscle mass is potentiated by the presence of other amino acids such as those contained in NUTRALYS® pea protein [12]. Therefore, pea protein could contribute to muscle protein synthesis when taken immediately after an effort. Considered globally, these arguments suggest that pea proteins ingestion might maximize muscle mass gains during resistance training. The aim of the present study was therefore to compare the effects of pea proteins against a reference protein (namely whey proteins which has previously been shown beneficial for muscle mass after resistance training [13,14]) and against placebo. It was hypothesized that pea proteins would be as efficient as the reference protein to increase both muscle thickness and strength.

## Methods

### Participants – ethics statement

A total of 161 male participants were recruited for the study. All had moderate physical activities (2–6 hours per week). None were engaged in any physical activity aimed at increasing muscle strength and mass for the six months before the experiment. All were healthy and free of injury during the three months preceding the study. The study excluded subjects who had asthma with potentially steroids treatment, consumption of high-protein diet, steroids treatment, current consumption of drugs or during the previous month, consumption of dietary supplement, sports drink, special dietary food or functional food, of any kind, liable or presented as liable to enhance physical performances and especially to increase muscle mass. Moreover, subjects with known hypersensitivity to any of the constituents of the studied products were excluded. Throughout the study, subjects maintained their usual training routine and diet. All gave their written consent after being carefully informed about the experimental protocol. The study was conducted in accordance with the Helsinki Declaration without any deviation from the protocol approved by the East I ethics committee (East I committee, France, number: 2011–47, 9 November 2011). The authors confirm that all ongoing and related trials for this intervention are registered before at the French agency for the safety of health products (AFSSAPS number: 2011-A01211-40) and at ClinicalTrials.gov (NCT02128516).

After inclusion, participants were randomly divided into three experimental groups: Pea (n = 53), Whey (n = 54), and Placebo (n = 54). Table 1 sets out their characteristics. Product randomization was balanced by block sizes of 10. The randomization code was not made available to anyone involved in conducting or evaluating the study and was released after the blind review and the freezing of the final database. The sample size was calculated *a priori* using Nquery Advisor software (version 6.01) based on the primary criterion (muscle thickness) and allowing for a power >90%. This statistical analysis indicated a minimum of 34 participants per experimental group.

**Table 1:** **Subjects characteristic at inclusion**

	**Pea**	**Whey**	**Placebo**	**Anova (** ***P*** **)**
Age (years)	22.0 ± 3.5	22.1 ± 3.6	21.7 ± 3.9	0.860
BMI (kg/m^2^)	23.1 ± 2.6	23.0 ± 3.0	22.9 ± 2.5	0.946
Biceps brachii thickness (mm)	25.0 ± 3.6	24.3 ± 3.8	25.4 ± 3.8	0.281
Mean circ. at rest (cm)	32.3 ± 2.5	32.0 ± 3.2	32.2 ± 2.5	0.829
Mean circ. contracted (cm)	33.3 ± 2.6	32.7 ± 3.0	32.9 ± 2.5	0.458
Isometric torque (N.m)	80.8 ± 14.1	78.5 ± 18.3	79.0 ± 14.9	0.731
Concentric torque (N.m)	62.7 ± 12.4	61.8 ± 14.3	64.0 ± 13.4	0.687
Eccentric torque (N.m)	92.3 ± 15.0	88.4 ± 17.8	88.9 ± 16.3	0.425
Arm-curl 1-RM (kg)	27.0 ± 6.3	25.3 ± 5.2	26.6 ± 5.3	0.260

BMI: body mass index; 1-RM: one maximum repetition; circ.: circumference.

### Experimental procedure

**Figure 1:**
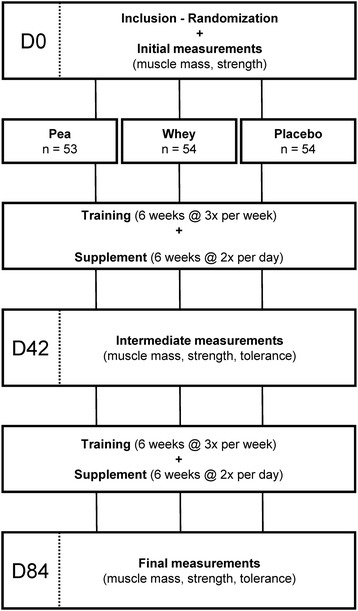
**Illustration of the experimental procedure.**

The objectives of this randomized, double-blind study conducted with parallel arms, was to evaluate the effects of oral Pea protein supplementation, versus Placebo and versus Whey proteins associated with a 12-week resistance training program, on elbow flexors muscle thickness (main outcome) and muscle strength (secondary outcome). The trial consisted in an inclusion visit (D0), an intermediate 6-week follow-up visit (D42) and a final 12-week visit (D84) (Figure 1). Visits were separated by weight training periods with three sessions per week. Testing sessions were conducted on non-training days and always at the same time of the day for the same subject. D0, D42 and D84 sessions included measurements of (i) muscle thickness using ultrasonography, (ii) arm circumference and (iii) maximal muscle strength in isokinetic conditions (concentric, isometric and eccentric). After the initial evaluation (D0), each subject was given a batch of products, according to randomization, and began weight training for a 12-week period. The same tests were repeated, in the same order, half-way through and at the end of the training period (D42 and D84, respectively). Tolerance, collected from adverse events and compliance with product intake (determined by counting products not consumed) was evaluated at D42 and D84 too.

### Training

All subjects followed the same training routine, three times per week with a rest day between each session. Training was based on three exercises involving the elbow flexor and extensor muscles. The exercises soliciting the flexor muscles were arm curl and lateral pull-down. In the arm curl exercise, subjects sat with weights in their hands with a ~40° trunk/arm angle. They had to flex/extend the forearm over the arm. For the lateral pull-down, subjects sat with a bar in their hands above the head. They had to flex/extend the forearm over the arm with a vertical movement. The exercise soliciting the extensors was the bench press. Subjects were lying on their backs with a bar in their hands with a 90° trunk/arm angle, arms extended, and had to flex and extend their upper limbs vertically. Throughout the training program, the number of sets was progressively increased from 2 to 5 while the number of repetitions was reduced in parallel from 15 to 5 repetitions maximum (RM). The final week, training was composed of three sets of 5 RM in order to preclude any fatigue for the D84 tests. Recovery between sets was 2–3 minutes. The load used for each exercise was regularly adapted during training depending on individuals’ maximum load (1-RM, one maximum repetition, evaluated every two weeks).

### Dietary supplementation

The three products under study were presented as 45 g sachets of banana-flavored cocoa powder to be diluted in 300 mL of cold water at each intake. The diluted drinks were identical in appearance, texture and taste and were isoenergetic (Table 2). Products were taken twice a day for 12 weeks. On training days, one dose was taken in the morning and the second just after training. On non-training days, one dose was taken in the morning and the second dose in the afternoon. The general food intake was not monitored over the experimental procedure but participants were instructed to maintain their diet habits throughout the experimental protocol.

**Table 2:** **Nutritional composition of drinks for 100 g of powder**

	**Placebo**	**Pea**	**Whey**
Energy value (kcal)	367	387	366
Proteins (g)	3.7	59.2	57
[of which pea or whey protein]	[−]	[55.6]	[53.2]
Carbohydrates (g)	82.5	21.0	20.2
Lipids (g)	1.5	6.3	4.9
Fibres (g)	4.4	5.1	6.7

A dose of powder contained either 25 g of vegetable Pea protein isolate (NUTRALYS®) or 25 g of Whey protein concentrate. The placebo, with no added protein, was composed of maltodextrin. The nutritional composition of each product and the amino acids content of Pea and Whey proteins are shown in Tables 2 and 3, respectively. The other components (fat-reduced cocoa, flavouring, aspartame, salt, silica dioxide) were identical in nature and in quantity in all three products. NUTRALYS® pea protein (ROQUETTE, Lestrem, France) is a vegetable protein isolate from the yellow pea (*Pisum sativum*). Peas are cleaned and ground in a dry process to produce pea flour. Flour is then hydrated and the pea starch and internal fiber are extracted separately. The protein fraction is then coagulated for further purification and, finally, carefully dried in a multi-stage spray dryer. The resulting highly purified pea protein isolate contains 85% protein, 7% fat, 3% carbohydrate, and 5% ash on a dry matter basis.

**Table 3:** **Amino acid composition (g) for 100 g of pea protein or Whey protein**

	**Pea**	**Whey**
Alanine	3.3	4.1
Arginine	6.6	2.1
Aspartic acid	8.9	8.7
Cystine	0.8	1.9
Glutamic acid	13.2	13.9
Glycine	3.1	1.5
Histidine	1.9	1.5
Isoleucine	3.7	4.9
Leucine	6.4	8.6
Lysine	5.7	7.2
Methionine	0.8	1.6
Phenylalanine	4.2	2.6
Proline	3.4	4.7
Serine	3.9	4.2
Threonine	2.8	5.7
Tryptophan	0.7	1.5
Tyrosine	3.1	2.8
Valine	4.0	4.6

### Measurements

#### Biceps brachii muscle thickness

Right-side biceps brachii muscle thickness was measured in real time using an ultrasound machine (AU5; Esaote Biomedica, Florence, Italy) coupled to a 50 mm probe at a frequency of 7.5 MHz. Subjects were lying supine with arms and legs completely relaxed. The right upper limb was positioned supine with a 45° angle with respect to the trunk. The probe was placed perpendicular to the skin surface at two-thirds of the distance between the acromion process of the scapula and the lateral epicondyle of the humerus [15]. The probe was coated with a water-soluble transmission gel to provide acoustic contact without depressing the dermal surface. Thickness was calculated as the distance between superficial and deep aponeuroses measured at the ends and middle of each 3.8 cm-wide sonograph. Three images were independently obtained for each point. The average value of these nine measures was calculated. To favor reproducibility, probe placement was carefully noted for reproduction during the other test sessions and measurements were always performed by the same operator.

#### Arm circumference

The circumference of the right arm was measured using a constant tension tape during maximal elbow extension at rest and during a maximal voluntary contraction (with maximal elbow flexion). Three measurements were made (at rest and contracted) along the length of the biceps, namely ¼, ½ and ¾ of the length of the upper arm (distance between the acromion process of the scapula and the lateral epicondyle of the humerus). Averaging was performed to obtain mean values for the circumference at rest and contracted.

#### Maximal voluntary torque

The maximal voluntary torque was measured on a Biodex (Biodex, Shirley, USA) isokinetic dynamometer during isometric, concentric and eccentric elbow flexions. The right-hand side was tested. Subjects were seated upright with a 95° hip angle. The upper limb was placed horizontally with the elbow rotation axis coinciding with the axis of rotation of the ergometer and aligned with the shoulder axis. The chest, shoulder and forearm were firmly attached to avoid perturbing contributions. Movements were made in the horizontal plan through a 120° elbow range of motion (from 10 to 130°, 0° = full extension). After a standardized warm-up consisting of submaximal contractions, measurements were made in concentric and eccentric conditions at an angular velocity of 60°.s^−1^. Subject had to accelerate or resist the ergometer lever arm, respectively. Five maximal voluntary contractions were performed consecutively for each condition. In isometric condition, the position was set at 80° elbow flexion and the subject had to produce a maximal voluntary contraction lasting 5 s. Three isometric contractions were requested with 60 s rest between contractions. Isometric, concentric and eccentric solicitations were presented in a random order and separated by five minutes of passive recovery. These various parameters were recorded for further analysis. The maximum value for each condition was retained for the statistical analysis.

#### Arm curl 1-RM

The maximum load (1-RM in kg) that could be lifted during elbow flexions was measured during an arm curl movement performed with both arms. For this, the load was progressively increased through successive sets (the first set being considered as warm up). Then, subjects were requested to lift each load only once. Care was taken to lift the load with the largest range of motion (~100 °). One minute rest was permitted between trials. In case of failure, a second try was allowed. The maximal load lifted was considered as the 1-RM. It was regularly evaluated (every two weeks) in order to adjust resistance training intensity.

### Statistical analyses

**Figure 2:**
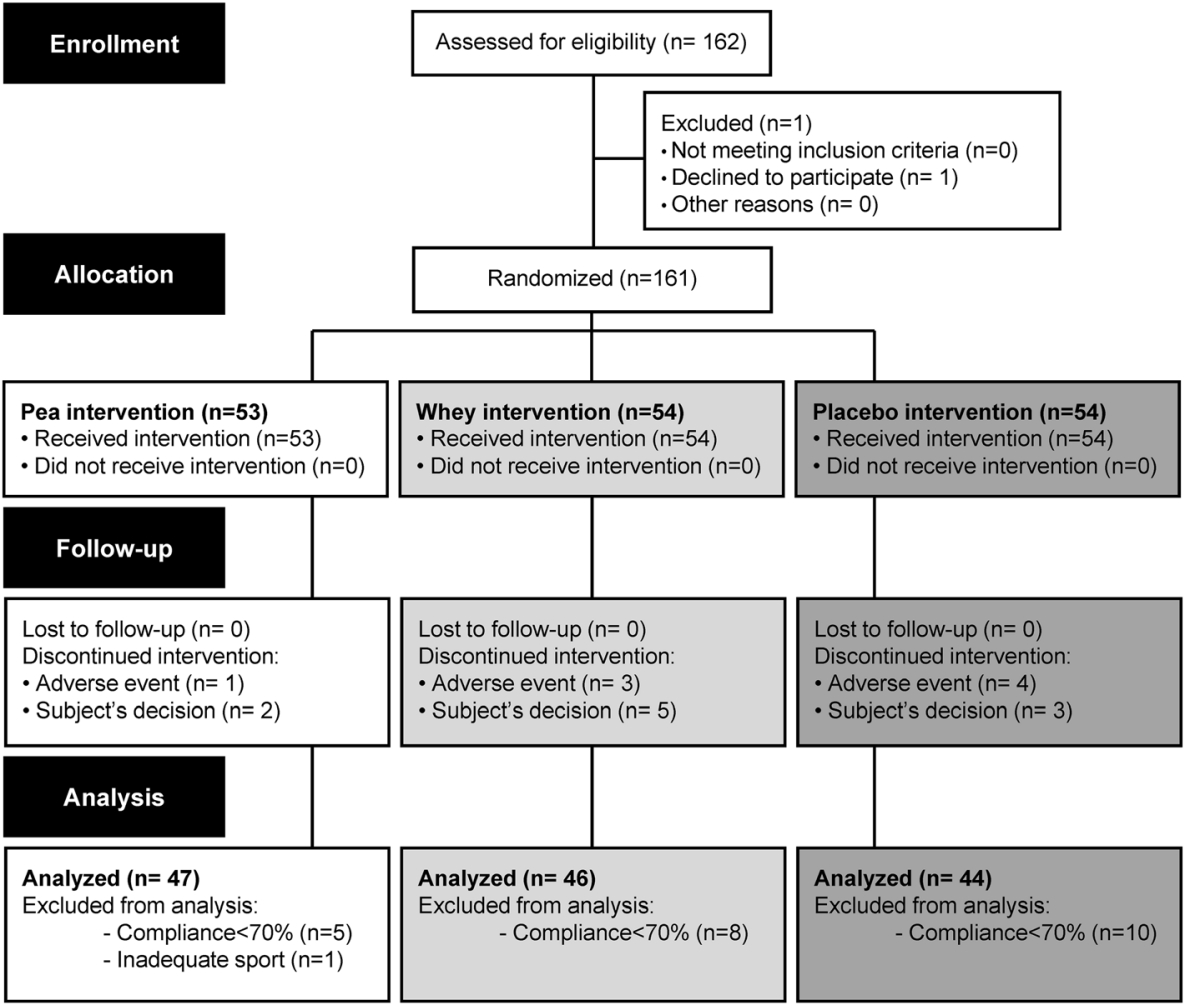
**CONSORT diagram outlining participants’ inclusion and drop out.**

Twenty four subjects left the study early due to personal reasons. At the end of the experimental procedure, 137 subjects were considered for analysis with 47 in the Pea group, 46 in the Whey group and 44 in the Placebo group (see Figure 2 for the CONSORT Diagram). Quantitative variables were presented as mean values and standard deviations. Values were tested using a repeated measures analysis of variances (ANOVA). Groups were used as independent variables and time (D0, D42, and D84) was used as the repeated variable. A sensitivity analysis was also conducted and considered subjects with a 1-RM at inclusion <25 kg (median value of study sample). Sixty eight subjects were considered for this sensitivity analysis. In the case of significant main effects or interactions, Scheffé post-hoc tests were conducted. Qualitative variables (supplementation compliance or adverse effects) were presented as absolute and relative frequencies and were tested by using a Chi square test. Statistics were conducted using SAS software (Ver. 9.2, SAS Institute, Inc., Cary, NC). *P* < 0.05 was taken as the level of statistical significance for all procedures.

## Results

### General observations

Initial values measured at D0 revealed similar groups for all variables (Table 1). During the experimental protocol, compliance was evaluated through the percentage of products returned by subjects. High average and comparable compliance was observed between groups: 93.4%, 90.8% and 90.7% for Whey, Placebo and Pea groups, respectively (*P* = 0.509). In addition, tolerance to the three products under study was good and comparable in terms of frequency and nature. Of the 161 subjects who took products at least once, three presented an adverse event in the Whey group (7.4%), four in the Placebo group (7.4%) and one in the Pea group (1.9%, *P* = 0.370). Except for two digestive disorders (type diarrhea) in the Placebo group, the adverse effects were all musculotendinous or back pains related to their usual activity throughout the study. All disappeared spontaneously except for an elbow tendinopathy in the Whey group which persisted at the end of the trial but any connection with the product was ruled out.

### Muscle thickness and circumference

**Figure 3:**
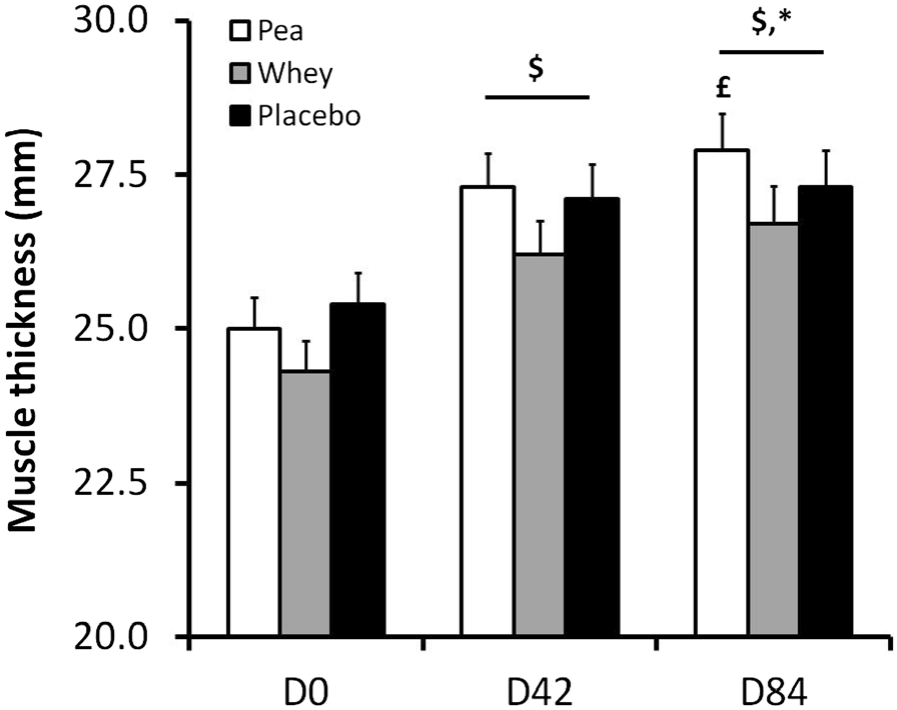
**Changes in biceps brachii thickness (mm) during the experimental protocol.** $: Significant difference within each group compared with D0 (*P* < 0.0001). £: Tending towards significance compared with D42 for the Pea group only (*P* = 0.09). *: Between group comparison between D0 and D84 approaching significance (*P* = 0.09).

**Figure 4:**
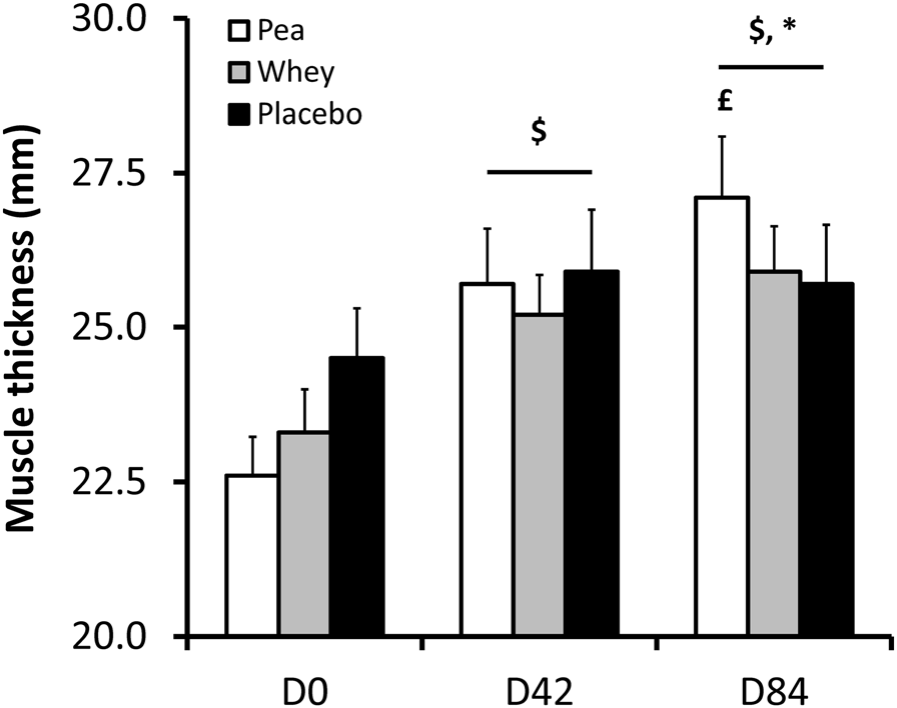
**Sensitivity analysis for biceps brachii thickness (mm) during the experimental protocol.** Data represent subjects with the 1-RM performance <25 kg at D0. Samples sizes are n = 17, 31 and 20 for the Pea, Whey and Placebo groups, respectively. $: Significant difference within each group compared with D0 (*P* < 0.05 – *P* < 0.0001). £: Significant difference compared with D42 for the Pea group only (*P* < 0.05). *: Between group comparison between D0 and D84 (*P* < 0.05).

Results showed a significant time effect for biceps brachii muscle thickness (*P* < 0.0001). Thickness progressively increases with time within each group (Figure 3). Neither group effect nor interaction was obtained. However, when comparing groups, relative increase between D0 and D84 tended towards statistical significance (+15.3 ± 12.7%, +13.4 ± 10.8% and +10.7 ± 8.6% for Whey, Pea and Placebo, respectively; *P* = 0.09). A sensitivity analysis, performed on the weakest participants on muscle thickness increase, highlighted a significant time effect (*P* < 0.0001) and interaction (group × time, *P* < 0.01). Thickness increases between D0 and D84 were +20.2 ± 12.3%, +15.6 ± 13.5%, and +8.6 ± 7.3% for Pea, Whey and Placebo, respectively. A Scheffé test showed a statistically significant difference between Pea and Placebo (absolute difference of the means 2.51 mm IC 95% (0.49; 4.53)) whereas there was no significant difference between Whey and Pea (absolute difference of the means 1.21 mm IC 95% (−0.63; 3.06)) nor between Whey and Placebo (absolute difference of the means 1.29 mm IC 95% (−0.46; 3.05)) (Figure 4).

Mid-training, differences between groups were not statistically significant (Figure 4). Moreover, it can be noted that the increase in the Placebo group (+8.8 ± 7.6% at D42 with respect to D0) was merely maintained with the six additional weeks of training (+8.6 ± 7.3% at D84 with respect to D0), whereas it further significantly increased in the Whey (from +10.7 ± 13.3% to +15.6 ± 13.5%) and Pea (from +13.6 ± 9.0% to +20.2 ± 12.3%) groups.

Changes in the right arm circumference at rest and during maximal contractions showed significant time effect (*P* < 0.0001) and increases over the 12-week period. The average circumference of the right arm at rest increased from 32.0 ± 2.3 cm at D0 to 32.4 ± 2.2 cm at D84 in the Placebo group (*P* = 0.005), from 31.6 ± 3.2 cm to 32.1 ± 3.2 cm in the Whey group (*P* = 0.0003) and from 32.3 ± 2.5 cm to 32.7 ± 2.5 cm in the Pea group (*P* = 0.01). The average circumference of the right arm contracted increased from 32.7 ± 2.2 cm (D0) to 33.7 ± 2.2 cm (D84) in the control group (*P* < 0.0001), from 32.4 ± 2.9 cm to 33.4 ± 3.2 cm in the Whey group (*P* < 0.0001) and from 33.3 ± 2.6 cm to 34.1 ± 2.4 cm in the Pea group (*P* < 0.0001). No difference was observed between the three groups at the end of the trial.

### Muscle strength

Maximal load (1-RM) during arm curl and muscle torque during the maximum voluntary isometric, concentric and eccentric contractions increased within each group. Statistical analyses only revealed a significant time effect (*P* < 0.0001). For example, for the Placebo group, the 12-week period produced an increase in the maximal 1-RM strength (+46.1 ± 22.4%), the maximal isometric (+20.5 ± 14.3%), concentric (+15.3 ± 16.2%) and eccentric (+17.2 ± 12.5%) torque. No significant group effect and interaction (group × time) was observed. For example, the increase in the maximum concentric torque between D0 and D84 was +8.8 ± 8.9 N.m for the Placebo group, +10.9 ± 9.9 N.m for Whey group and +10.7 ± 7.6 N.m for Pea group.

## Discussion

The present study aimed to test the hypothesis that a supplementation with pea protein, used in association with resistance training, would be as efficient as Whey protein to increase muscle thickness and strength. The present results showed significant gains in muscle mass as attested by thickness of the biceps brachii in all groups. Differences between groups were observed with the sensitivity study considering participants with the lowest muscle force at the entrance in the protocol. Pea protein group displayed a significantly greater effect than the Placebo on muscle thickness and Whey protein occupied an intermediate position between the other two supplements.

The results obtained on biceps brachii showed an increased muscle thickness with Pea protein. The effects obtained, although greater than those for Whey, do not reach the statistically significant level. The absence of statistical superiority over Whey is not a counter performance for pea protein which is positioned as an alternative to Whey, which, on the contrary, fails in this study to reach the statistical significance level against the Placebo group. Whey was used as a benchmark because most studies have reported a positive effect on muscle hypertrophy in various populations such as young athletes [16-18]. This hypertrophy is supposed to be due to activation of the mTOR (mammalian target of rapamycin) signaling pathway resulting from the simultaneous action of protein ingestion and training [17]. The almost similar supplementation effect of Pea and Whey may be ascribed to the characteristics of both ingredients. Both beverages are particularly rich in essential BCAA (valine, isoleucine and leucine) which play an important role in muscle protein synthesis. A literature review [19] indicated that leucine reportedly stimulates the muscle protein synthesis required to replace muscle protein damaged by resistance exercises. In young adults, protein synthesis is 20% higher after ingestion of leucine, proteins and carbohydrates, compared with ingestion of carbohydrates and proteins with no leucine intake [19]. Moreover, authors reported that consumption of 8 to 11.5 g of EAA containing 2 to 3 g of leucine after exercise may maximize the protein synthetic response [20-22]. In the present study, such content is provided.

The lack of statistical difference between NUTRALYS® and Whey may be attributed to the quite similar amino acids content but also to the kinetic of digestion. Whey protein has a fast kinetic of digestion, bringing rapidly high concentration of amino-acids in plasma after ingestion, but this effect is transient and returns to resting levels within 2-3 h [7]. NUTRALYS® is an intermediate profile fast protein (unpublished observations) and it can be assumed that the amino acid content in blood plasma would increase quickly after ingestion, making it readily and long lastingly available in the body to participate in muscle protein synthesis. In addition, based on Protein Digestibility Acid Corrected Amino Acid Score [23], NUTRALYS® pea protein has shown that it is a high nutritional quality protein with an index of 92.8% out of a maximum 100% [24] corresponding to the values of Whey or casein, while fruit proteins have a mean value of 76% and cereal proteins 59% [25].

Each experimental group underwent 12 weeks of resistance training. As shown previously [26] and as attested by the Placebo group, weight training alone had an impact on biceps brachii thickness. This increase in muscle thickness was obtained during the first six weeks of training. Such result is in general accordance with the literature since muscle architectural changes have been shown to occur between the fourth and eighth weeks of resistance training [27]. Interestingly, beyond six weeks of training, only the groups taking protein supplementations, either Pea or Whey experienced additional increases in muscle thickness. This result suggests that, for a long training period (> six weeks), the association of resistance training and protein consumption is important. It will maintain a positive protein balance (ratio of protein synthesis to degradation in favor of synthesis) and therefore muscle hypertrophy [28].

An increase in muscle thickness, observed here, is fundamental for achieving a gain in strength [29]. However, muscle strength, although improved after the experimental period, was not different between groups. Such result is quite surprising since protein beverages have exacerbated muscle thickness increases and as a consequence should have increased muscle strength gains as compared with the placebo. This lack of difference in muscle strength between groups remains unclear but could be attributed to several factors such as the supplementation characteristics, training type and training status.

Protein was supplemented twice with an intake immediately after the resistance training session. Although debated, such timing may appear as one of the most effective nutrient timing strategies for muscle protein synthesis [11]. As compared to a morning/evening intake group, Cribb and Hayes [30] observed larger muscle mass increases with protein intake before and after training. The best stimulus for protein synthesis appeared to be with protein feeding in close proximity to training sessions [31,32], with feeding recommended within the first two hours post-exercise [2,33,34].

Protein quantity was 50 g.day^−1^ with 25 g after resistance sessions. These doses seem unlikely to be responsible for the lack of difference between groups for muscle strength. Indeed, in a recent review [35], the author recommended the ingestion of 20 g of high quality protein immediately after exercise to maximally stimulate protein synthesis. Therefore, sufficient protein intakes have been proposed here and are likely not responsible for the lack of differences between groups for muscle strength.

It should be remembered that training and supplementation effects are potentiated in subjects exhibiting lower muscle strength at inclusion (1-RM < 25 kg, sensitivity analysis). Such a result is not surprising since training is well known to have larger effects in untrained subjects. For example, greater increases in muscle cross-sectional area have been reported in subjects who had not previously engaged in resistance training in comparison with more accustomed subjects [36]. The effects of amino acids supply may also depend on training status, since greater disturbances in protein turnover (protein synthesis and degradation) are obtained following training in novice than in experienced athletes [37]. Moreover, the expected increase in protein synthesis following exercise appears to be smaller and shorter in trained athletes as compared with untrained subjects [38,39]. Thus, training status may influence muscle performance. Indeed, Vieillevoye et al. [10] found increases in lower body strength with an EAA supplement while no modification was obtained with placebo. Surprisingly, in the same study, strength was similarly enhanced in both groups for the upper body. These authors concluded that supplementation and training adaptations seem to depend on the initial training status; the weaker the subjects, the larger the effect of protein supplementation on muscle strength. Moreover, the present study was conducted in physically active males. It is possible to speculate that, with untrained participants, differences between groups might have been revealed more easily. Furthermore, a plateau, or ‘ceiling effect’ , of the adaptive responses to training is generally observed either for strength gains and muscle protein synthetic response [37,40]. Hence, protein requirements and training stimulus are affected by training status and duration. For instance, greater protein intakes are required during the early stages of intensive bodybuilding training and more particularly in novices [41]. Modification of the training program might also have exacerbated differences between groups for all studied parameters. Training volume [42] concomitant with the load used in terms of 1-RM’s percentage [43] are possible parameters.

## Conclusions

The present experiment demonstrated that protein supplementation may exacerbate possible adaptations induced by resistance training. The consumption of pea protein promotes gains in biceps brachii thickness and especially in beginners or people returning to weight training. This statistical superiority compared with the Placebo and the comparable results with those obtained for Whey intake make pea protein an alternative to Whey-based dietary products for athletes from different levels and sports. Such proteins should also be of interest in other populations such as elderly to slow down the aging process and maintain muscle mass.

## Abbreviations

BCAA: Branched-chain amino acids
EAA: Essential amino-acids
D0: Testing at inclusion
D42: Testing in the middle
D84: Testing at the end
RM: Repetition maximum
